# Adolescent development of context-dependent stimulus-reward association memory and its neural correlates

**DOI:** 10.3389/fnhum.2015.00581

**Published:** 2015-10-28

**Authors:** Joel L. Voss, Jonathan T. O’Neil, Maria Kharitonova, Margaret J. Briggs-Gowan, Lauren S. Wakschlag

**Affiliations:** ^1^Department of Medical Social Sciences, Northwestern University Feinberg School of MedicineChicago, IL, USA; ^2^Ken and Ruth Davee Department of Neurology and Interdepartmental Neuroscience Program, Northwestern University Feinberg School of MedicineChicago, IL, USA; ^3^Department of Psychiatry, University of Connecticut Health CenterFarmington, CT, USA; ^4^Institute for Policy Research, Northwestern UniversityEvanston, IL, USA

**Keywords:** regulation, hippocampus, prefrontal cortex, basal ganglia, reinforcement learning, reversal learning

## Abstract

Expression of learned stimulus-reward associations based on context is essential for regulation of behavior to meet situational demands. Contextual regulation improves during development, although the developmental progression of relevant neural and cognitive processes is not fully specified. We therefore measured neural correlates of flexible, contextual expression of stimulus-reward associations in pre/early-adolescent children (ages 9–13 years) and young adults (ages 19–22 years). After reinforcement learning using standard parameters, a contextual reversal manipulation was used whereby contextual cues indicated that stimulus-reward associations were the same as previously reinforced for some trials (consistent trials) or were reversed on other trials (inconsistent trials). Subjects were thus required to respond according to original stimulus-reward associations vs. reversed associations based on trial-specific contextual cues. Children and young adults did not differ in reinforcement learning or in relevant functional magnetic resonance imaging (fMRI) correlates. In contrast, adults outperformed children during contextual reversal, with better performance specifically for inconsistent trials. fMRI signals corresponding to this selective advantage included greater activity in lateral prefrontal cortex (LPFC), hippocampus, and dorsal striatum for young adults relative to children. Flexible expression of stimulus-reward associations based on context thus improves via adolescent development, as does recruitment of brain regions involved in reward learning and contextual expression of memory.

**Highlights**
- Early-adolescent children and young adults were equivalent in reinforcement learning.
- Adults outperformed children in contextual expression of stimulus-reward associations.
- Adult advantages correlated with increased activity of relevant brain regions.
- Specific neurocognitive developmental changes support better contextual regulation.

## Introduction

One crucial function of memory is the use of past experience in the guidance of ongoing behavior to meet current situational demands (Wang et al., 2015). Contextual cues present in the environment thus must be used to determine the contents of memory that are relevant to performance in the current situation. This type of flexible expression of memory based on context has been associated with interactivity of distinct brain regions, particularly hippocampus and prefrontal cortex (Preston and Eichenbaum, 2013). For instance, activity of rodent hippocampal neurons can signal memory for an object at each of two learned locations, and a contextual cue can trigger which of these two representations are expressed on a given retrieval trial (Navawongse and Eichenbaum, 2013). Temporary inactivation of prefrontal cortex eliminates appropriate contextual expression of the activity patterns without disrupting the activity patterns *per se* (Navawongse and Eichenbaum, 2013), showing that it is the interactivity of prefrontal cortex with hippocampus that allows appropriate flexible expression of memory based on context. In primates, lateral prefrontal cortex (LPFC) and dorsal striatum comprise a strongly interconnected network (Alexander et al., 1986), and similar contextual expression of memory likely depends on hippocampus in conjunction with a network involving both prefrontal cortex and dorsal striatum (Wang and Voss, 2014). Given that these regions and their interconnections develop heavily during the adolescent years (Ghetti and Bunge, 2012), we aimed to better understand their contributions to contextual memory expression by identifying developmental changes in their activity during a context-dependent stimulus-reward association memory task.

The protracted development of prefrontal cortex has been linked to a similarly slow trajectory of improvement in various executive functions throughout adolescence (Bunge and Zelazo, 2006). For instance, set-switching abilities represent one form of flexible expression of knowledge associated with prefrontal cortex, particularly LPFC (Best and Miller, 2010). Set-shifting and similar abilities likely do not reach full maturity until late adolescence or early adulthood (Bunge and Zelazo, 2006; Best and Miller, 2010), when prefrontal cortex is relatively well developed. However, LPFC development does not occur in a vacuum, and developmental improvements in memory abilities likely depend on changes in LPFC as well as hippocampus and dorsal striatum, and on the structural interconnectivity of these regions (e.g., Ghetti and Bunge, 2012). Indeed, some evidence indicates that hippocampal recruitment for memory-related processing is not similar to that in adults until at least approximately 14 years of age (Ghetti et al., 2010). Furthermore, hippocampal and LPFC interaction continues to develop throughout adolescence (Finn et al., 2010; Ghetti and Bunge, 2012).

A developmental trajectory likewise has been observed for performance in tasks that depend on dorsal striatum, such as reinforcement and reversal learning (Luking et al., 2014). Developmental increases occur in the ability to learn the associations between specific stimuli and either rewards or punishments in reinforcement learning tasks based on feedback (Crone et al., 2004; Baldwin et al., 2012). Performance differences between children/early-adolescents and adults are less apparent when reinforcements are consistent rather than probabilistic (Eppinger et al., 2009; Hämmerer et al., 2011). Indeed, some have found no differences in reinforcement learning when reinforcements are 100% consistent (Shephard et al., 2014). Reliable differences can be identified between children and adults in reversal learning (Crone et al., 2004; Eppinger et al., 2009; Hämmerer et al., 2011; Koolschijn et al., 2011) even when performance in the acquisition phase is matched due to 100% consistency (Shephard et al., 2014). However, reversal is normally studied as an isolated event (i.e., all stimulus-reward associations are learned, and then they reverse, with learning the reversal measured as latency to acquiring the new reversed associations). In contrast, contextual expression of memory to support adaptive behavior must occur in response to discrete contextual cues (Navawongse and Eichenbaum, 2013; Preston and Eichenbaum, 2013; Wang and Voss, 2014). That is, studies of contextual memory expression require that the subject have two distinct representations of learned stimulus-stimulus or stimulus-response associations in memory which can be selected for expression based on specific sensory cues, whereas set-shifting and reversal learning tasks focus on the ability to update behavioral rules or stimulus-response associations when presented with repeated feedback. Thus, it is currently unclear the extent to which developmental changes occur in flexible context-dependent expression of memory and whether they are similar to observed developmental changes in set-shifting and reversal-learning abilities.

We developed a novel context-dependent association task to identify developmental effects on the contextual expression of stimulus-reward associations based on trial-level contextual cues. Subjects first learned stimulus-reward pairings using 100% consistency of reinforcement. Then, during a context-dependent reversal phase, subjects were tested such that stimulus-reward associations were consistent with learning on some trials (consistent trials) but reversed on others (inconsistent trials), as indicated by a contextual cue (the side of the screen on which stimuli were presented). This task was performed during functional magnetic resonance imaging (fMRI) scanning by pre/early-adolescent children and young adults. We predicted that children and adults would demonstrate roughly equivalent performance for the acquisition phase, given that 100% consistent reinforcement was used. In contrast, we predicted one of two patterns during the context-dependent reversal phase: (1) children could be non-specifically impaired relative to young adults, showing impairments for both consistent and inconsistent trials reflecting poor general contextual regulation ability or (2) children could have specific problems with the inconsistent trials due to the selective demand for flexible expression of memory required on these trials. Furthermore, we used fMRI to identify neural correlates of correct performance, hypothesizing that activity of LPFC, hippocampus, and dorsal striatum would be associated with context-dependent reversal learning and would differ between early-adolescents and adults.

## Materials and Methods

Data were collected and analyzed from pre/early-adolescent children (*n* = 14; 8 female, ages 9–13 years at testing, mean age = 10.5 years) and young adults (*n* = 14; 7 female; ages 19–22 years at testing, mean age = 21.4 years). An additional three children and two young adults participated, but their data were excluded due to excessive movement during fMRI scanning (two excluded subjects, see below) and partial datasets (three excluded subjects). All subjects or their legal guardians provided informed written consent and all study procedures were approved by the Northwestern University Institutional Review Board. Subjects were free from reported neurological, psychiatric, and developmental conditions and were not currently taking psychoactive medications (using self-report for adult subjects and parent-report for children).

Subjects performed a novel reward-learning task that involved contextual reversal. The task involved a series of six two-phase blocks. Three of the blocks involved a rigged frustration condition that is not analyzed for the current report, and so data from the three blocks that did not involve rigged frustration are reported. Different stimuli were used in each of these three two-phase blocks.

**Figure 1:**
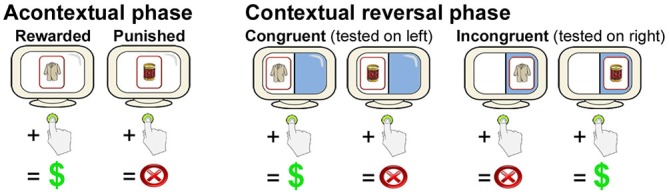
**Overview of the contextual reversal-learning task.** Subjects learned stimulus-response associations via feedback for stimuli presented at the center of the screen during the Acontextual phase. During the contextual phase, stimuli were presented on either the right or the left of the screen. Stimulus-response associations were the same as during the Acontextual phase when presented on the left (congruent trials) and were reversed when presented on the right (incongruent trials). Location of testing was randomized across trials. Only two cards (of four in each Acontextual-Contextual experiment block) are shown.

During the first phase of each of the three blocks, subjects learned acontextual stimulus-reward associations via feedback. For each trial, one of four cards each depicting a nameable object (Rossion and Pourtois, 2001) appeared at central fixation for 1800 ms each, followed by 2200-ms response periods (during which a “?” appeared at fixation), and then a pseudorandomized ISI period during which a fixation cross was present at central fixation. Subjects were required to press a button to select a card or to refrain from pressing the button to avoid selecting a card. Two of the cards were consistently associated with a 10-point reward/gain when selected, whereas two of the cards were consistently associated with a 10-point punishment/loss when selected. There was no loss or gain when subjects refrained from pushing the button (and therefore no feedback). Subjects were instructed that winning points would provide additional payment at the end of the experiment. Subjects learned via feedback that was provided immediately after a 2200-ms response period (see Figure 1). There were 24 trials, divided equally among the four stimuli, which were presented in pseudorandomized order with a pseudorandomized ISI of 2000–8000 ms (mean = 6000 ms).

During the second phase of each of the three blocks, subjects performed the contextual reversal-learning task. The same four cards used during the first (acontextual) phase were used. The visual display was divided in half by a vertical line with distinct background colors for the left vs. right half of the display (Figure 1). On each trial, a card could appear either on the left or the right side of the screen. As for the first phase, subjects pushed a button or refrained from pushing in response to each stimulus. When cards appeared on the left side of the screen, stimulus-response mappings were congruent with those learned during study (i.e., the same two stimuli were rewarding and the same two stimuli were punishing). However, when cards appeared on the right side of the screen, stimulus-response mappings were incongruent with study (i.e., cards that were originally rewarding were punishing and cards that were originally punishing were rewarding). Thus, subjects had to reverse learned stimulus-response associations for right-side presentations and maintain originally learned stimulus-response associations for left-side presentations. Each of the four cards was presented six times in the congruent condition and six times in the incongruent condition, for 48 trials total (half congruent and half incongruent, half rewarding and half punished), presented in pseudorandomized order. Different stimuli were used in each of the three two-phase blocks. Trial timing, including stimulus duration, response period duration, and ISIs, was identical as to the first phase.

The primary performance measure was *d*^′^, a normalized value of correct responses to rewarded items (hits) minus incorrect responses to non-rewarded items (false alarms). Raw hit and false alarm rates are reported in Table 1.

**Table 1:** **Summary of response accuracy for acontextual and contextual phases**.

	Rewarded hit	Non-rewarded false alarm	Response time rewarded hit
**Acontextual phase**			
Children	0.956 (0.012)	0.008 (0.002)	712 (24)*
Adults	0.969 (0.018)	0.027 (0.016)	620 (30)*
**Contextual phase: Congruent trials**
Children	0.981 (0.011)	0.056 (0.012)	809 (19)**
Adults	0.978 (0.018)	0.024 (0.017)	674 (42)**
**Contextual phase: Incongruent trials**
Children	0.915 (0.031)	0.147 (0.031)**	845 (22)**
Adults	0.971 (0.019)	0.050 (0.012)**	683 (40)**

Mean accuracy values are provided (proportion correct) for Hits to rewarded stimuli and False Alarms to punished stimuli. Mean response times are also given. *P < 0.05 and **P < 0.01 vs. corresponding conditions for pre/early-adolescent children vs. young adults. Parentheses indicate SEM.

Before fMRI scanning, subjects first practiced a non-computerized version of the task outside of the scanner that used printed cards and coins. This helped ensure that children understood the general structure of the task. Then, subjects performed one practice two-phase block in a mock MRI scanner using different stimuli as in the scanned blocks. All subjects demonstrated comprehension of the task prior to fMRI scanning (no subjects were excluded due to failure of comprehension).

MRI data were collected using a Siemens 3T TIM TRIO scanner with a 32-channel head coil. Visual stimuli were back-projected onto a screen and viewed through a mirror attached to the head coil. The projected display subtended 18.8° of visual angle vertically and 23.1° horizontally, with a resolution of 1250 by 1024 pixels and a refresh rate of 60 Hz. Visual stimuli subtended approximately 11.5° by 8.5° of visual angle. Whole-brain BOLD EPI was collected with AC-PC alignment during task performance (TR = 2000 ms, TE = 20 MS, voxel size = 1.72 × 1.72 × 3 mm, FOV = 768 × 720, Flip angle = 80°). Acontexual phases of each block included 86 volumes (2 min 54 s) and contextual phases of each block included 138 volumes (4 min 38 s). A structural image was acquired following task performance to provide anatomical location (MPRAGE T_1_-weighted scans, TR = 2300 ms, TE = 3.41, voxel size = 1-mm^3^, FOV = 25.6 cm, flip angle = 8°, 176 sagittal slices). Responses were made using an MRI compliant button box.

MRI data were analyzed using the AFNI software package (Cox, 1996). Preprocessing steps included motion correction, slice timing correction to the first slice, functional/structural coregistration, stereotactic transformation using Montreal Neurological Institute (MNI) 305 template, resampling to 1.5 mm^3^ isotropic voxels, and spatial smoothing with a 4-mm FWHM Gaussian Kernel. Two subjects were excluded from analyses because >15% of volumes across all functional runs were marked as having >3 mm or >3° of estimated motion in any direction. Event-related activity estimates were derived using a deconvolution approach within a GLM. Trials were modeled as a regressor of event onsets using a boxcar function of 3-s duration convolved with a canonical hemodynamic response function. T_1_ and T_0_ components of the MRI signal and 6-parameter motion estimates were entered as nuisance variables. A different model was used for the contextual and acontextual phases of each block. The conditions that were modeled included correct responses (or non-responses) for rewarded and non-rewarded stimuli during the acontextual phase (trials with incorrect responses or non-responses for both stimulus categories were modeled as a separate condition that was not analyzed), as well as correct responses or non-responses for congruent and incongruent rewarded and non-rewarded stimuli for the contextual phases (trials with incorrect responses or non-responses for all stimulus categories were modeled as a separate condition that was not analyzed).

Group-level effects were identified using whole-brain voxel-wise *t*-tests or repeated measures ANOVA (RM-ANOVA). All analyses concerned activity differences for correct responses to rewarded stimuli, as correct non-responses to non-rewarded stimuli cannot be as readily interpreted (i.e., either successful inhibition of response or temporary lack of attention). Furthermore, we focused on correct responses to rewarded stimuli because hit rates did not vary by age group or condition. In contrast, false alarm rates to non-rewarded stimuli varied by age and congruency conditions (see “Results” Section), and so age-related differences in neural correlates for non-response trials could be due merely to different performance levels. For the acontextual phases, a group-level *t*-test was thus performed to compare activity for trials with rewarded stimuli and correct responses for the child vs. young-adult groups. For the contextual phases, RM-ANOVA was used including the factors group (child or young-adult) and condition (trials with correct responses to rewarded congruent and incongruent stimuli). For all analyses, a voxel-wise threshold of *P* < 0.005 was used in combination with a cluster-size correction of 57.4 mm^3^ determined by Monte Carlo simulation with AFNI program 3dAlphaSim to yield a corrected *P* < 0.05.

## Results

### Acontextual Reinforcement Learning

Pre/early-adolescent children and young adults learned stimulus-response associations during acontextual phases with high accuracy (mean *d*^′^ = 4.59 and 4.44, respectively, *t*_(13)_ = 17.4, *P* < 0.0001 vs. zero (chance performance) for children and *t*_(13)_ = 16.4, *P* < 0.0001 vs. zero for young adults). The difference in *d*^′^ between groups was not significant (*t*_(26)_ = 0.4, *P* = 0.69). However, there was a significant difference in mean response times, with responses made faster by adults than by children (Table 1; *t*_(26)_ = 2.4, *P* = 0.023).

Paralleling the lack of differences in *d*^′^ between groups, there were no age-related differences in neural correlates of correct performance for the acontextual phase. The whole-brain voxel-wise *t*-test comparing activity associated with correct responses to rewarded stimuli for children vs. adults did not identify any significant activity differences.

### Contextual Reversal Learning

**Figure 2:**
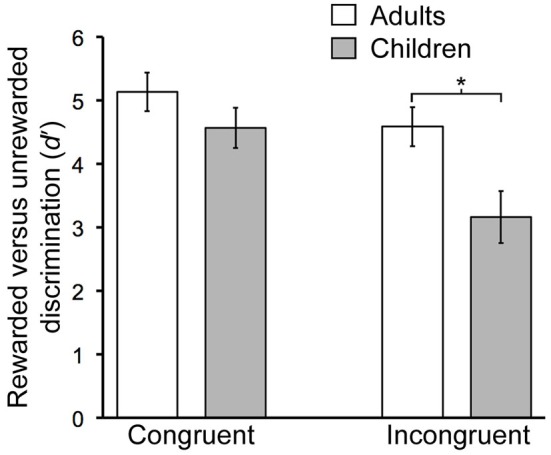
**Developmental differences in contextual memory.** Mean discrimination sensitivity (*d^′^*) values are shown for Congruent and Incongruent trial types during the Contextual phase. The age-by-condition interaction was significant (see text). Error bars indicate SEM. *P = 0.01.

There were significant age-related performance differences for the contextual reversal phase (Figure 2). RM-ANOVA for *d*^′^ scores with factors group (child/young-adult) and condition (congruent/incongruent) indicated a significant main effect of group (*F*_(1,26)_ = 6.06, *P* = 0.021), as well as significant interaction of group by condition (*F*_(1,26)_ = 14.76, *P* < 0.001). *Post hoc* pairwise comparisons indicated significantly lower *d*^′^ values for children vs. adults for the incongruent condition (*t*_(26)_ = 2.8, *P* = 0.01) but not for the congruent condition (*t*_(26)_ = 1.3, *P* = 0.21). Thus, during contextual reversal, age-related performance differences were selective for incongruent trials. As indicated in Table 1, this group *d*^′^ difference was due to significantly more false alarm responses made by children than by adults in the incongruent condition, with no significant group differences in hit rates.

There was a main effect of age group on response times, with faster responses for adults than for children (*F*_(1,26)_ = 10.88, *P* = 0.003), and a main effect of trial type, with faster responses for congruent than incongruent trials (*F*_(1,26)_ = 5.87, *P* = 0.023). The interaction of age group with trial type was not significant (*F*_(1,26)_ = 2.09, *P* = 0.16).

**Figure 3:**
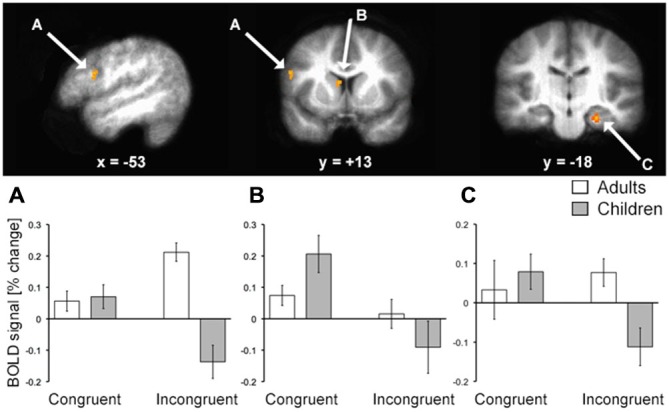
**fMRI activation corresponding to developmental differences in contextual memory.** fMRI activity corresponding to the interaction of age group (pre/early-adolescent children vs. young-adult) by condition (Congruent vs. Incongruent trial types) for trials with correct responses to rewarded stimuli is shown superimposed on the average brain of all subjects in stereotactic space. Parameter estimates for each condition of interest are shown in each of the three primary regions identified. Note that interaction terms are significant at *P* < 0.005 by definition (based on the whole-brain voxel-wise threshold) and pairwise *P* values are not shown to avoid redundant statistical information. **(A)** LPFC; **(B)** dorsal striatum; **(C)** hippocampus.

To identify fMRI activity corresponding to the age-related *d*^′^ differences, we first focused on the group-by-condition interaction using whole-brain voxel-wise RM-ANOVA. In alignment with our *a priori* hypotheses regarding relevant brain regions, this analysis identified activity of LPFC, hippocampus, and the body/head of the caudate (Figure 3; Table 2). Activity of these regions for correct responses to rewarded items thus differed significantly by age group and by congruency. Estimated activity for these regions was extracted in order to identify whether the significant interaction term reflected activity differences that mirrored behavioral performance differences (i.e., no marked age differences for congruent trials and clear differences for incongruent trials). As shown in Figure 3, this was the case for LPFC and hippocampus. In contrast, the interaction identified for the caudate body/head was due to relatively greater activity for children than adults in the congruent condition and *vice versa* for incongruent condition. Activity of other regions was also identified by the interaction analysis, including several regions of posterior cingulate cortex and ventral visual cortex (Table 2), but follow-up analyses were not performed, as we had no *a priori* hypotheses regarding these regions. It is important to note that there was no interaction of age group by condition in response times, and so interaction effects on neural activity were not secondary to effects on response times.

**Table 2:** **Summary of fMRI findings**.

			Centroid coordinates		
	Volume	Side	*X*	*Y*	*Z*	BA
**Interaction of age by condition**
Inferior frontal gyrus*	84.4	L	−53	+15	+18	44/45
Caudate body/head*	64.1	L	−8	+13	+8	–
Hippocampus body/head*	60.8	R	+26	−19	−25	–
Precuneus posterior cingulate gyrus	175.5	R	+8	−53	+45	7/31
Lingual gyrus	70.9	L	−11	−79	−6	18
Posterior cingulate gyrus	60.8	R	+15	−41	+10	29
Posterior cingulate gyrus	60.8	L	−4	−37	+27	23
**Main effect of age**
Middle occipital gyrus	587.3	R	+41	−79	+20	39
Inferior parietal lobule	259.9	L	−32	−39	44	40
Middle occipital gyrus	131.6	L	−40	−88	+9	19
Middle occipital gyrus	108.0	L	−36	−81	+20	19
Middle temporal gyrus	87.8	L	−53	−65	+1	37
Middle occipital gyrus	84.4	R	+15	−94	+18	18
Angular gyrus	74.3	R	+31	−58	+36	39
Inferior parietal lobule	74.3	L	−58	−30	+38	40
Superior parietal lobule	60.8	L	−32	−60	+49	7

Including volume (mm^3^), hemisphere/side (Left or Right), coordinates of the centroid voxel in MNI-305 space, and Brodmann’s Areas (BA). *Regions shown in Figure 3 based on a priori hypotheses.

Although our primary hypotheses concerned fMRI activity reflecting the interaction of age group by congruency condition, we also assessed the main effects of age group. Significant age-related differences in activity were identified by the main effect analysis for age, whereby adults had greater activity relative to children in a collection of regions including posterior hippocampus, lateral PFC, ventral striatum, and ventral visual cortex (Table 2). The behavioral ramifications of this increased activity are unclear, as age-related differences in *d*^′^ were specific to incongruent trials. Greater non-specific activity for young adults relative to children could potentially reflect a combination of factors, including increased automatic encoding of task information (e.g., Ghetti and Bunge, 2012; increased visual attention, more efficient neural processing, and the overall difference in response times that was identified between groups). Indeed, non-specific age-based increases in task-related activity in these regions has been identified in other studies (Klingberg et al., 2002; Kwon et al., 2002; Thomason et al., 2009; Kharitonova et al., 2015).

## Discussion

The primary finding of this experiment is that pre/early-adolescent children demonstrated specific impairments of contextual reversal and corresponding neural activity relative to young adults. Despite performing as well as young adults in general (acontextual) reward learning and showing no differences in fMRI activity associated with reward learning, children performed worse during contextual reversal and demonstrated reductions in neural activity compared to adults. These differences were specific in that children performed equally to adults for congruent trials during the contextual reversal phase, demonstrating impairment selectively on incongruent trials (which were randomly intermixed). Thus, contextual reversal was not non-specifically disruptive for children (i.e., worse performance for all trials within the phase), but rather had specific disruptive consequences when stimulus-reward associations had to be flexibly expressed in an incongruent fashion on a subset of trials.

Neural correlates of successful performance differed for children vs. adults with similar specificity for incongruent trials during the contextual reversal phase. Activity of LPFC and hippocampus demonstrated matched activity for congruent trials but differed between age groups for incongruent trials (as identified by significant age-by-condition interaction and *post hoc* tests). For both regions, activity for adults was greater than activity for children on incongruent trials, suggesting greater recruitment of these regions in adults to support task performance. For dorsal striatum (caudate body/head), there was also an age-by-condition interaction, but the activity pattern was different than for LPFC and hippocampus. Here, the interaction reflected relatively greater activity for children compared to adults in the congruent condition and relatively less activity for children in the incongruent condition (without significant pairwise differences). It is possible that this activity pattern reflected the relative difference in reward between the congruent and incongruent conditions that was more pronounced for children than for adults. That is, because performance was lower for children than young adults in the incongruent condition, the relative difference in reward value for congruent vs. incongruent trials would have been higher for children (i.e., greater perceived reward given a relatively lower baseline reward level across the task), and neurons throughout the dorsal (and ventral) striatum can signal relative rather than absolute reward value (e.g., Cromwell et al., 2005). Indeed, one potential limitation of our study is that reward feedback always followed behavioral responses, and so brain activity cannot be attributed to decision-making vs. reward and feedback processing. It is also possible that the dorsal striatum findings might reflect lower levels of presynaptic dopamine (Matthews et al., 2013), which is critical for flexible gating of information into working memory (Atallah et al., 2004). Overall, these fMRI findings indicate distinction between dorsal striatum and LPFC/hippocampus contributions to contextual memory expression, with LPFC/hippocampus demonstrating similar activity patterns that are more tightly linked to the behavioral expression of contextual memory than dorsal striatum.

These results replicate one previous finding that 100% reinforcement consistency leads to matched performance in reward learning for early-adolescent children vs. adults (Shephard et al., 2014), which is broadly consistent with many findings that developmental effects on reinforcement learning are reduced as reinforcement consistency approaches 100% (Eppinger et al., 2009; Hämmerer et al., 2011). Developmental differences could have further been reduced by the relatively advanced age of our early-adolescent sample. Nonetheless, children differed significantly from adults when reversal demands were required, which is consistent with a wealth of previous findings that reversal is more highly problematic for children than reinforcement learning. Our findings show that performance is not non-specifically disrupted during the reversal phase, but rather that disruption occurs specifically when reversal must be expressed. That is, in our paradigm, consistent and inconsistent trials were intermixed, and behavioral and neural differences were specific to inconsistent. Thus, development specifically improves regulation, and this can be observed even when regulation needs are intermixed with non-regulation needs.

The LPFC region with activity that differentiated incongruent trials for children vs. adults corresponds approximately to the left inferior region associated with verbal working memory in youth and adults (Kwon et al., 2002) and that is active during controlled memory retrieval (Barch et al., 2001; Badre and Wagner, 2007). Adults might have maintained both sets of stimulus-response associations in the contextual phase better than children, whereas children might have perseverated on the learned associations. However, this explanation is unlikely given that behavioral differences were mainly evident for false alarm responses to inconsistent items (Table 1). That is, if children merely perseverated by expressing consistent stimulus-response associations for inconsistent trials, then both hits and false alarms would have differed. Another possibility is that children could have responded reactively rather than proactively to stimuli to a greater extent than adults (Braver et al., 2009; Chatham et al., 2009), and the neural activity differences in LPFC and hippocampus for children vs. adults could have reflected the ramifications of differences in proactive response planning. Indeed, LPFC-hippocampal interactions have been previously reviewed in the context of proactive planning of behavioral responses (Wang et al., 2015).

These effects could be considered as reflecting working memory, in the context of flexible/contextual expression of memory representations (Miller and Cohen, 2001). Interestingly, a previous study (Finn et al., 2010) found increased hippocampal activity across adolescent development in an area closely matching that identified here (Figure 3) in a simple delay working-memory task. This was interpreted as greater hippocampal recruitment to solve working memory problems by adolescents with the slower emergence of prefrontal involvement that was more pronounced later in development. This finding is exactly contrary to ours, which shows greater hippocampal recruitment for young adults relative to children. This could be due to the fact that our task is not a simple delay-response working memory task, but rather requires flexible expression of memory. Indeed, flexible responding in tests of working memory such as the Wisconsin Card Sorting task is critically dependent on the hippocampus in adults (Gupta et al., 2009). This is evidence in favor of distinctions of short-term memory (working memory maintenance) from working memory in the sense of flexible/contextual responding, which could have different developmental trajectories. Flexible responding likely is more heavily dependent on long-term memory retrieval and joint contributions from LPFC and hippocampus (Miller and Cohen, 2001; Navawongse and Eichenbaum, 2013), and slower to emerge with development.

To summarize, the current findings help elucidate the developmental trajectory of flexible memory expression during adolescence. This essential function is not fully operational until at least the young-adult period, given that pre/early-adolescent children in our study performed worse than young adults and exhibited reduced recruitment of critical brain regions, including LPFC, hippocampus, and dorsal striatum. Further, these developmental differences did not reflect global impairment induced by reversal demands, given that children differed in behavior and brain activity from adults only for inconsistent trials during the reversal phase. Future research should address limitations of the current design, which includes inability to fully separate decision-making from reward-related neural processing, inability to pinpoint cognitive operations engaged during incongruent trials by children vs. adults, and a relatively small cross-sectional sample. Longitudinal measurement will be needed to track the developmental emergence of flexible memory abilities supported by the brain regions described here across adolescent development.

## Conflict of Interest Statement

The authors declare that the research was conducted in the absence of any commercial or financial relationships that could be construed as a potential conflict of interest.
